# Surgical Therapy of Congenital Nevi in the First Year of Life—Psychological Impact on Parents

**DOI:** 10.1177/12034754231199750

**Published:** 2023-09-21

**Authors:** Lena Schwenk, Johanna Graf, Katrin Kofler, Hans-Martin Häfner, Lukas Kofler

**Affiliations:** 1 9188 Department of Dermatology, University Medical Center, Eberhard Karls University, Tuebingen, Germany; 2 7979 Internal Medicine VI Department of Psychosomatic Medicine and Psychotherapy, University Hospital Tübingen, Tübingen, Germany; 3 576395 Psychooncology Section, Comprehensive Cancer Center Tübingen-Stuttgart, University Hospital Tübingen, Tübingen, Germany; 4 Center for Skin Diseases skin+more MVZ, Biberach a.d.R., Germany; 5 Center for Rare Skin Diseases, Eberhard Karls University, Tuebingen, Germany

**Keywords:** congenital naevus, dermatosurgery, psychological burden

## Abstract

**Background:**

The birth of a child with a congenital melanocytic nevus (CMN) can lead to distress in the parents. Surgical treatment of CMN can begin in infancy.

**Objectives:**

To provide insight into the perspective of parents of children with CMN regarding their experienced psychological burden and motivation to undergo surgery as well as their satisfaction.

**Methods:**

Retrospective analysis of patient data of infants (< 1 year of age), who were surgically treated by power stretching for CMN using subcutaneous infiltration anesthesia (SIA) from 01/01/2020 to 08/31/2021. To evaluate the parent’s motivation and psychological burden during the surgical treatment of their child, a questionnaire was designed to interview them in a standardized telephone-based interview.

**Results:**

Out of 45 interviewed parents, 62.2% indicated “severe” or “very severe” distress at the time of their child’s birth. Distress was mostly reduced by information about diagnosis and treatment (n = 34) and treatment-progress (n = 27). Stigmatization was experienced by 35.6% of parents. 84,5% of parents were highly satisfied with early initiation of surgical therapy. 69% felt “very satisfied” or “satisfied” with the outcome of surgery. Motivation for surgical therapy was concern about malignant transformation of the, possible stigmatization of the child due to the nevus, while most of the parents (73.3%) mentioned both.

**Conclusions:**

Surgical treatment of CMN by power stretching in SIA in infancy is associated with high levels of satisfaction among the children’s parents. Early initiation of surgical therapy and education about the diagnosis can reduce the psychological burden of the parents and can prevent psychosocial problems in affected children.

## Introduction

A nevus corresponds to a hamartomatous skin malformation.^1,2^ Congenital melanocytic naevi (CMN) are benign proliferations of nevoid melanocytes that usually appear on the skin at birth.^1,3^ Depending on the size, small, medium and large as well as giant congenital nevi are distinguished. Some classifications also include the number of satellite nevi to provide a more standardized data collection and estimation for the probability of occurrence of adverse events, such as melanoma.

Congenital melanocytic nevi of all sizes occur in approximately 1% of newborns^3^ with a slightly higher prevalence in female neonates.^4^ Typically, the size is estimated as projected adult size. Giant congenital nevi can cover a large portion of the body’s surface. They most commonly occur on the trunks.^5,6^ The occurrence of satellite nevi has been observed in 52.7% to 78% of patients.^7,8^

Several studies have attempted to determine the risk of melanoma in patients with congenital nevi, showing that no blanket statement can be made in this regard, as the risk of melanoma occurrence in patients with congenital melanocytic nevi depends on the extent of the nevus, its location, and number of satellite nevi.^9,10^ Krengel et al. showed that the risk of melanoma depends on the size of the congenital nevus and that patients with giant congenital nevi with a diameter of ≥40 cm have a much higher risk for the occurrence of melanoma.^10^ In addition to the risk of malignant degeneration, stigmatization as well as psychological effects in patients with congenital melanocytic nevi are a relevant indication for therapy. Children with large and giant congenital nevi have an increased incidence of emotional, social, and behavioral problems.^11-13^ In particular, an increase in social problems at school entry, such as withdrawal or aggressive behavior, have been observed.^12,13^ Studies also show that the disease can have a negative impact on the quality of life of affected children.^12,14^ This is especially true when congenital melanocytic nevi are associated with health problems such as pruritus or neurological symptoms, and affected individuals feel severe stigma due to congenital melanocytic nevus.^12^ Neuhaus et al. showed that the quality of life of affected individuals was more impaired in adolescence than in childhood.^14^ Stigmatization is particularly experienced by affected individuals with nevi on highly visible areas of the skin such as the face and hands.^14^ Patients who were treated surgically before the age of 3 years mostly had no negative memories of stigmatization associated with the nevus.^15^

Whether and how congenital melanocytic nevi should be treated is discussed controversially. Arad et al. suggest that the indication for surgical therapy should be made especially in patients with high-risk nevi, since in these signs of malignant transformation are more difficult to detect. As such, they define giant nevi, nevi localized to the trunk, and those that have an inhomogeneous surface structure. Changes in the appearance of the nevus e.g., the forming of proliferative nodules are harder to detect in these high risk nevi due to the size and their inhomogeneous surface structure. In addition, surgical therapy should be considered if nevus leads to functional impairment.^16^ The compelling indication for surgical therapy of a congenital melanocytic nevus arises in cases of malignant transformation.^2^ Serial excisions and defect closure by power stretching allow the resection of large nevus areas and complete resection of large and giant congenital melanocytic nevi.^17^ The data show that early surgical removal of a nevi means less psychological impact and better quality of life for patients. Therefore, psychological distress must be taken into account in the treatment planning and it is often a main reason for surgical treatment at an early age of the patients. It has been shown, that a large proportion of affected children prefer a scar to the original nevus in appearance for aesthetic reasons, as they feel that the scar is more socially acceptable.^11,15,18^ Nevertheless, surgical therapy in infancy means massive distress and uncertainty for parents regarding this treatment planning. It was shown that a good preparation for surgery and education of parents caused a significant stress reduction and parents were able to improve decision making for the therapy planning.^19^

The aim of this study was to shed light on the views of parents of children with congenital melanocytic nevi, especially in relation to surgical therapy. It was important to gain a better understanding of their decision-making regarding surgical therapy and the associated psychological burdens. In addition, the handling of the diagnosis in general was investigated.

## Methods

### Patient Data and Study Design

A retrospective analysis of patient data of infants who were surgically treated for congenital melanocytic nevus at the Department of Dermatology, University of Tübingen, Germany was performed. Children who were surgically treated for congenital nevus using subcutaneous infiltration anesthesia (SIA) during the study period (01/01/2020, 08/31/2021) and were less than 1 year old at that time were included. The indication for surgical treatment was made individually if a CMN was present and the parents desired treatment. All parents were informed about therapy options including regular observation without surgical therapy.

The data collected included sex, patient age at the time of the first surgical intervention, number of surgical interventions in SIA within the study period, and the sessions over which the operations were distributed. In addition, the following peri- and postoperative complications were collected: wound infection, postoperative bleeding, suture dehiscence, wound healing disorder, hematoma, and pain. The neonatal infant pain scale (NIPS) score was used to evaluate the occurrence of pain. The criteria for scoring were facial expression, breathing, posture of the arms, posture of the legs, and state of mind with 0 or 1 point, and crying with 0-2 points.

Furthermore, the localization of congenital nevi in each child was determined. This was simplified according to the head/neck, trunk, upper extremity, and lower extremity locations. For each surgically treated nevus in a child, the size of the resected area in square millimeters (mm²) per surgical procedure and the amount of tumescent solution applied during the surgical procedure in milliliters (ml) were collected.

Exclusion criteria for the present study were as follows: age >1 year at the time of the first surgery, surgery under general anesthesia, and other skin tumors except melanocytic nevi.

### Surgical Treatment Procedure

An experienced surgeon first conducts a detailed educational discussion about the diagnosis and possible treatment options for every child presented to our hospital. If the parents decided to undergo surgical treatment, excision of the nevus was aimed at the third month of life of the child’s life. Serial excisions are typically planned in children with congenital giant nevi. When possible, multiple sessions were planned between the third and sixth month of life to achieve the greatest possible reduction of the nevus area within the first year of life.

All procedures were performed under local anesthesia within the first year of life. For this purpose, local anesthesia using SIA was used. The tumescent solution used for infants consisted of 10 ml lidocaine 2%, 10 ml ropivacaine 1%, and 0.5 mg epinephrine 1:1000 in whole electrolyte solution.^20,21^ The tumescent solution was slowly dispersed into the subcutaneous tissue after injection. Excisions were spindle-shaped. Following excision, defect closure was performed using intracutaneous butterfly sutures, according to the technique described by Breuninger et al..^22^ Suture strips were then applied along the entire length of the suture to relieve pressure on the wound edges. Finally, pressure dressing was applied.

### Calculation of the Resected Nevus Area

The length and width of the resected spindles were recorded in the surgical reports. To calculate the resected area, the shape of an ellipse was assumed instead of a spindle shape.

The resected area (A) is thus calculated from the two semi-axes a and b (in mm) and the circle number π using the formula A = a * b * π

### Questionnaire

To evaluate the experiences and psychological impact of parents of children with congenital melanocytic nevi, a questionnaire was designed to interview the parents in a standardized telephone-based interview. It was covering the following topics: subjective burden of the parents, stigmatization and therapy satisfaction. The questionnaire used was not validated or standardized. The interview was performed once in 12/2021.

Some of the questions are exemplary mentioned below:

How much did you feel burdened by the diagnosis of your child at the time of birth?

How much do you feel burdened because of your child’s disease at this moment?

If you feel less burdened now, which factors did contribute to that?

Why did you seek surgical treatment for your child’s nevus?

Did you/your child experience stigmatization because of the congenital melanocytic nevus?

In the case of the numerical rating scales, 0 points were classified as “not at all,” 1-3 points as “a little,” 4-6 points as “moderately,” 7-9 points as “strongly,” and 10 points as “very strongly.”

### Statistics

Data were recorded using Microsoft Excel. Statistical analysis and the creation of tables and diagrams were performed using Microsoft Excel.

### Ethical Approval

This study was approved by the Ethics Committee of the Medical Faculty of the Eberhard-Karls-University and University Hospital Tübingen (699/2021BO2).

## Results

45 of the 49 parents contacted participated in the telephone survey, with one parent answering each question asked. Thus, 45 patients and their parents were included in this study. This corresponds to 91.8% of contacted parents. The parents were interviewed at a median of 19 months after childbirth. A minimum of 5 months elapsed between the birth of the child and the interview with the parents, and a maximum of 30 months.

Patients were a median of 3 months old at the time of the first surgery, with a mean of 3.04 months. The youngest child was 1 month old at the start of treatment, while the oldest child was 7 months old at the time of the first surgery.

On a median, the study participants underwent three surgeries (maximum: 11 surgeries, minimum: 1 surgery). As some patients had nevi at different sites, the number of operations per patient refers to the total number of operations performed, however, they may have been performed at different sites. The median number of surgeries performed by the study participants was distributed over two surgical sessions.

Twenty-seven patients had nevi on the head and neck, 23 had nevi on the trunk, 7 had nevi on the upper extremity, and 14 had nevi on the lower extremity. All sites of nevi were documented, not only the main site where surgery was performed.

The first procedure resected a median of 1343.0 mm² nevus, the second procedure resected a median of 1378.4 mm² nevus, the third procedure resected a median of 1721.6 mm² nevus, the fourth procedure resected a median of 1669.0 mm² nevus, the fifth procedure resected a median of 2670.4 0 mm² nevus, and the sixth procedure resected a median of 1963.5 mm² nevus. The seventh and eighth procedures were performed in only one child included in the study and therefore no median values were calculated.

### Complications

No complications occurred during surgical treatment in 88.9% of patients. In 5 patients (11.1%), peri- and postoperative complications were documented; in 2 patients (4.4%), a wound infection developed, which required antibiotic treatment. One patient (2.2%) developed a suture dehiscence. One patient (2.2%) developed a wound healing disorder, defined as wound healing that did not proceed as expected according to the postoperative stage. Two patients (4.4%) experienced pain according to NIPS, requiring treatment in one case. No intraoperative or postoperative complications related to SIA were observed.

### Subjective Burden of the Parents

When asked about the degree of distress caused by the diagnosis of congenital melanocytic nevus immediately after the birth of their child, most parents (62.2%) indicated “severe” or “very severe” distress, 28.9% indicated “moderate” distress, and only 8.9% felt “little” distressed by the diagnosis at the time of the child’s birth.

At the time of the interview, none of the parents surveyed felt “very much” burdened by the child’s illness anymore. The extent of the burden caused by their child’s illness at the time of the survey was indicated as “strong” by 4.4% of the parents. 22.2% felt “moderately” burdened by their child’s illness at the time of the survey and 62.2% felt “little” burdened. 11.1% stated that they felt “not at all“ burdened.

For parents who indicated a ‘very strong’ burden at the time of birth (10 points), the numerical value with which the burden was indicated at the time of the survey was reduced by a median of 6.5 points. Parents who felt “strongly” burdened by their child’s illness at the time of birth (7, 9 points) gave a median value for the burden that was 4 points lower at the time of the survey. It is clear that parents who indicated a particularly strong distress (7, 10 points) at the time of the child’s birth experienced a particularly strong reduction in distress up to the time of the survey.

Parents who reported reduced subjective burden at the time of the survey compared to the time of their child’s birth were asked about the factors that contributed to the reduction in stress.

Most frequently, information about the diagnosis and possible treatment options (34 mentions) and the progress of treatment to date (27 mentions) contributed to the reduction in subjective distress. Subsequently, discussions with other affected parents, support from family and friends, and organization in a self-help group were mentioned with equal frequency, each with six mentions. Furthermore, getting used to the disease (three mentions) and one’s own research on the disease (one mention) were mentioned as stress-reducing factors.

### Motivation for Surgical Therapy

15.6% of the parents stated that the main reason for surgery was their concern about malignant degeneration of the nevus, while 11.1% stated concern about stigmatization as the sole reason. However, the majority of parents (73.3%) said that both reasons contributed to their decision to have surgery.

### Stigmatization

Of the parents interviewed, 35.6% stated that they or their children had experienced stigmatization due to congenital melanocytic nevus. A total of 64.4% of the participants had never experienced stigma at the time of the survey. Nearly half (48,1%) of parents of children with a congenital melanocytic nevus on the head or neck (n = 27) indicated that they had experienced stigmatization, whereas only 16,7% of the parents of children with nevus on other locations (n = 18) experienced stigmatization. As children included in the study were still infants or toddlers at the time of the survey (12/2021) and therefore were not able to report the level of stigmatization experienced, our study can only represent the experience and concerns of their parents.

### Therapy Satisfaction

In all children participating in the study, the start of surgical therapy occurred before completion of the first year of life. When asked about their satisfaction with the decision to start surgical therapy for congenital nevus early, the majority of parents (84.5%) stated that they were “very satisfied” or “satisfied” with the decision.

At 69%, the majority of parents are “very satisfied” or “satisfied” with the aesthetic and functional outcome of the surgical procedures at the time of the survey. 24.5% indicated “very satisfied,” 44.5% are “satisfied.” 20.0% of the respondents are “moderately satisfied,” 11.1% are “not very satisfied” with the treatment result. Since the therapy had not been completed by 73.3% of the children included in the study at the time of the survey, the assessment of the functional/aesthetic outcome refers, in most cases, to an intermediate status.

In retrospect, 43 of the 45 parents interviewed (95.6%) would decide again to undergo surgical treatment of their child’s congenital melanocytic nevus.

## Discussion

This study emphasizes that the birth of a child with congenital melanocytic nevus represents a great psychological burden for affected parents. Of the parents interviewed, 62.2% stated that they felt strongly to very strongly burdened by the diagnosis at the time of their child’s birth. In addition, 35.6% of the parents stated that they or their child had experienced stigmatization due to the nevus. At the time of the survey, which was at a median of 19 months after the birth of the child, the burden decreased significantly and the parents showed a high satisfaction with the surgical treatment.

Parents of children with congenital melanocytic nevi have so far received little attention in the literature. However, some results indirectly point to the burden of affected parents. For example, in their study of the psychosocial effects of surgical treatment for congenital melanocytic nevi, Bellier-Waast et al. showed that 26% of parents of children with congenital melanocytic nevi never took photographs of their child while they had a visible nevus to escape the confrontation with the diagnosis.^15^ Surgical therapy has the potential to reduce stigmatization of children with congenital melanocytic nevi. Further, it was shown that more than half of the children who had a congenital melanocytic nevus excised were either not ashamed of the scars or felt affected by them.^15^

The motivation of parents for surgical therapy were the concern about malignant development of the nevus, which was mentioned by 15.6% of the parents interviewed here as a reason for the decision to undergo surgical therapy, as well as the concern about a possible stigmatization of the child due to the nevus, which 11.1% of the parents interviewed stated as decisive for the motivation for surgical treatment in the foreground. In most of the parents interviewed (73.3%), the reasons overlapped. Other authors have shown a strong motivation for treatment, influenced by the hope of reducing the risk of melanoma. For example, in their study of 136 patients evaluating surgical treatment for congenital melanocytic nevus, Ceballos-Rodriguez et al. showed that the motivation of parents and patients surveyed for surgical treatment was hoped for a melanoma risk reduction of 28%, aesthetic considerations were the decision in 17%, and both of the above reasons in 55%.^18^

Concern about the stigmatization of the child due to congenital melanocytic nevus, which many parents interviewed cited as a motivation for surgical therapy, may be an important reason for the decision to undergo surgical therapy, especially in the case of congenital melanocytic nevi at sites that cannot be covered by clothing.

Overall, the results of the surgical treatment of congenital melanocytic nevi seem to meet the expectations of the affected parents. In our study, almost all parents interviewed would even recommend surgical therapy to other affected families, with a very high or high probability (97.8%).

The high percentage of parents voting for surgical therapy on their child’s nevus due to concerns of malignant transformation of the nevus indicates that the the risk of melanoma in CMN might be overestimated.^10^ Therefore, continuous is necessary, as regular clinical evaluation of the nevus a valid approach for patients with low risk CMN. Parents are individually informed that the color of a CMN can lighten over the course of time, typically determined by the skin color. Superficial removal techniques do not have the potential to influence this process.^23^ Excision of a CMN allows it to be histologically examined, whereas this is not possible with other procedures such as laser removal or dermabrasion.^10^

The power stretching technique allows excisions of large tissue parts without the need for suture removal, which can be bothersome or even painful for children.^24^ In addition, there is no need for painful stretching of the skin before the actual procedure as with expander techniques, nor is it necessary to harvest skin for grafting at a second site.

Limitations of our study are the lack of comparison of different treatment methods, as well as the correlation of nevus size and further follow-up. As this was not a controlled study, we cannot make any statements about the amount of reduction in subjective burden without surgical intervention. In addition, surgical treatment was only completed in 73.3% of the children at the time of the survey, which leads to a limited significance in regards to the satisfaction with the postoperative result.

## Conclusion

Surgical treatment of congenital nevi in SIA by power stretching in infancy is a treatment method with few complications, which is associated with a high level of satisfaction among the parents of the young patients interviewed in our study and seems to have the potential, among other contributing factors, to reduce psychosocial problems as stigmatization in the affected children.

Information about the diagnosis and classification of possible complications forms the basis for parents’ decision for or against surgical therapy and should therefore be provided early, in detail, and empathetically. The diagnosis usually comes as a complete surprise to parents at birth and represents a high level of distress. This burden can be reduced by education about the low risk of malignant degeneration of congenital nevi as well as by early initiation of surgical therapy.
